# Sexual Differences in Cell Loss during the Post-Hatch Development of Song Control Nuclei in the Bengalese Finch

**DOI:** 10.1371/journal.pone.0125802

**Published:** 2015-05-04

**Authors:** XiaoNing Chen, Jia Li, Lei Zeng, XueBo Zhang, XiaoHua Lu, MingXue Zuo, XinWen Zhang, ShaoJu Zeng

**Affiliations:** 1 Beijing Key Laboratory of Gene Resource and Molecular Development, Beijing Normal University, Beijing, China; 2 College of Life Sciences, Hainan Normal University, Haikou, China; Fudan Univeristy School of Pharmacy, CHINA

## Abstract

Birdsongs and the regions of their brain that control song exhibit obvious sexual differences. However, the mechanisms underlying these sexual dimorphisms remain unknown. To address this issue, we first examined apoptotic cells labeled with caspase-3 or TUNEL in Bengalese finch song control nuclei - the robust nucleus of the archopallium (RA), the lateral magnocellular nucleus of the anterior nidopallium (LMAN), the high vocal center (HVC) and Area X from post-hatch day (P) 15 to 120. Next, we investigated the expression dynamics of pro-apoptotic (Bid, Bad and Bax) and anti-apoptotic (Bcl-2 and Bcl-xL) genes in the aforementioned nuclei. Our results revealed that the female RA at P45 exhibited marked cell apoptosis, confirmed by low densities of Bcl-xL and Bcl-2. Both the male and female LMAN exhibited apoptotic peaks at P35 and P45, respectively, and the observed cell loss was more extensive in males. A corresponding sharp decrease in the density of Bcl-2 after P35 was observed in both sexes, and a greater density of Bid was noted at P45 in males. In addition, we observed that RA volume and the total number of BDNF-expressing cells decreased significantly after unilateral lesion of the LMAN or HVC (two areas that innervate the RA) and that greater numbers of RA-projecting cells were immunoreactive for BDNF in the LMAN than in the HVC. We reasoned that a decrease in the amount of BDNF transported via HVC afferent fibers might result in an increase in cell apoptosis in the female RA. Our data indicate that cell apoptosis resulting from different pro- and anti-apoptotic agents is involved in generating the differences between male and female song control nuclei.

## Introduction

Birdsong is one of the most complex vocal behaviors among non-human animals. Birdsong exhibits significant sex differences and therefore provides an excellent model for studying the neural mechanisms of sexual differentiation [1]. How are these sexually dimorphic structures established during development, and what are the cellular and neural mechanisms underlying their differentiation? To date, these questions are unanswered.

Birdsong is governed by a series of well-described, interconnected brain nuclei that form two distinct pathways [2–4]. The motor pathway is initially innervated by the HVC, then projects to the RA, and eventually innervates the tracheosyringeal portion of the hypoglossal nucleus (nXllts) [3]; The anterior forebrain pathway (AFP) begins in the HVC, then makes synaptic connections to Area X, the medial nucleus of the dorsolateral thalamus (DLM) and the LMAN before finally connecting with the RA [5–7]. The LMAN makes neural connections with the DLM and RA within approximately 15 days after hatching, while the HVC sends afferent inputs to the Area X or RA at approximately 25 days after hatching. Any lesion that leads to an incomplete motor pathway or anterior forebrain pathway will disturb normal song production [8].

Most song control nuclei are sexually dimorphic, but these dimorphisms are exhibited in different manners. First, sexual differences manifest in the occurrence of some nuclei, including the HVC and Area X. Before the critical period (around 30 days after hatching), the number of neurons produced in the ventricular zone is markedly greater in males than in females, and a greater number of neurons in males migrate to the HVC and Area X [2, 9–12]. Second, sexual differences do not occur within the critical period but rather gradually occur later. The RA is similar in males and females prior to the critical period, but the number of neurons, and its volume and size, decrease after the critical period to a much greater extent in females than in males [13, 14]. To our knowledge, the cellular (e.g., neuronal reduction through the classical apoptotic pathways) and neural mechanisms underlying the neuronal reduction observed in the RA remain unknown.

Tissue development depends on the finely tuned regulation of apoptosis, or programmed cell death [15]. Two major pathways are involved in apoptosis. One is the death receptor pathway, which is triggered by binding death ligands such as tumor necrosis factor (TNF). The receptors can recruit adapter proteins to their cytosolic death domains, which then bind death effector domains and caspase recruitment domains, resulting in the activation of effector protease caspase-3 and, finally, apoptosis. The other is the mitochondria-cytochrome C pathway [16–19]. Bcl-2 family members play critical roles in the mitochondria-cytochrome C pathway [20], but not in the death receptor pathway [16–19]. Some Bcl-2 family members, including the BH3-only (Bad and Bid) and Bax (Bax and Bak) subfamilies, can trigger mitochondrial protein release and thus exhibit pro-apoptotic activity, [21–23]. Unphosphorylated Bad localizes to the mitochondria and binds Bcl-2 and Bcl-xL to promote apoptosis. However, serine phosphorylated Bad localizes to cytosol and binds to 14-3-3 proteins rather than Bcl-2 or Bcl-xL, and does not induce apoptosis [24, 25]. Additionally, Cytc release is promoted by Bid upon cleavage into tBid by caspase-8 or by BAK through the disruption of outer mitochondrial membrane integrity [26]. In contrast, some other Bcl-2 family members, including Bcl-xl and Bcl-2, are anti-apoptotic proteins. Both Bcl-xl and Bcl-2 have been shown to maintain the mitochondrial membrane potential by blocking the translocation of Bax to the mitochondria [22, 24, 27–29].

Activated caspase-3 staining and terminal deoxyribonucleotide transferase dUTP nick-end labeling (TUNEL) are widely accepted methods for labeling apoptotic cells [30–33], and both have advantages and disadvantages [34–36]. To date, only a single report has failed to reveal differences in apoptosis between the male and female HVC and the overlying ventricular zone in which HVC progenitors are generated by TUNEL staining [37]. It is not currently known how apoptosis contributes to sexual differences in songbird song control nuclei or how Bcl-2 family members (both pro- and anti-apoptotic) are involved in apoptosis in these nuclei. Because apoptosis mediated by the mitochondria-Cytc pathway, not by the death receptor pathway, has been reported to be involved in neural tissue development [15], the death receptor pathway is not investigated in the present study.

To address the role of apoptosis in the sexual differentiation of song control nuclei, the present study first detected apoptotic cells in several song control nuclei (RA, LMAN, HVC and Area X), and examined the sex-related differences in these nuclei in the Bengalese finch (*Lonchura striata*) at post-hatching days (P) 15, 25, 35, 45 and 120 by caspase-3 immunohistochemistry. The presence of apoptotic cells was further confirmed by TUNEL staining in some experimental groups. We then studied the expression of proapoptotic (Bid, Bad and Bax) and anti-apoptotic (Bcl-2 and Bcl-xL) members in the aforementioned song control nuclei. Finally, we investigated whether the neural afferents from the HVC and LMAN (the only two nuclei that project to the RA) to the RA caused a reduction in RA size or in the number of RA neurons immunoreactive for brain-derived neurotrophic factor (BDNF) or its receptor tyrosine protein kinase B (TrkB). It has been shown that, following the binding of BDNF, released from both pre- and postsynaptic compartments, to the TrkB receptor, the ensuing signaling cascade converges on the mitogen-activated protein (MAP) kinase pathway through the activation of extracellular signal-regulated kinase (ERK) [38, 39]. Phosphorylated ERK then activates one or more targets including cAMP-response element binding protein (CREB), immediate early genes, cytoskeletal elements, genes involved in protein synthesis, and voltage- and ligand-gated ion channels, resulting in neuron survival and synapse growth or plasticity [40–43].

Our results revealed significant sexual differences in the number of apoptotic cells in the RA and LMAN at P45 that corresponded to differential patterns of pro-apoptotic and anti-apoptotic gene expression. RA volume and the total number of BDNF- and TrkB-expressing neurons decreased to greater extents following electrical lesion of the LMAN compared to the HVC at P18–22.

## Materials and Methods

### Animals and tissue preparation

The Bengalese finches (*Lonchura striata*) used in our study were purchased from a local supplier (Beijing Guanyuan Flowers and Birds Market, Beijing Haidian District, Beijing, China) and raised in a breeding colony at Beijing Normal University. The birds were maintained in cages of standard size (50 cm×62 cm×38 cm) in a room under a 14/10 h light/dark cycle at 20–30°C. Each cage contained 4–7 birds and was equipped with perching sites and nest boxes. Seed and fresh water were provided at all times, and green vegetable supplements were provided occasionally. All experiments in our study were conducted in accordance with the guidelines of the Beijing Animal Protection Committee. Siblings were raised with their parents in groups of two to five. The birds were divided into five age groups that included juveniles (P15, 25, 35 and 45) and adults (>90 days) (n = 4–6 for each experimental group). The birds were anesthetized via intramuscular injection of 20% barbiturate (Sigma, 50 μl/g body weight) and then perfused with cold 0.9% saline and 4% paraformaldehyde in 0.1 M phosphate buffer (pH 7.4). The brains were separated and fixed for 24 h in the same fixative at 4°C. The brains were then transferred into 30% sucrose at 4°C and stored overnight or until they sunk to the bottom. The hemispheres were then cut into 10 or 40 μm sagittal slices with a freezing microtome (CM 1850, Leica). A total of five to seven sets of sections were collected for each brain. The sections were stored at -20°C until use.

### Immunohistochemistry

The 10 μm sections were used for caspase-3, Bcl-xL, Bax, BDNF and TrkB immunohistochemistry. The sections were first incubated in 3% H_2_O_2_ for 15 min to quench endogenous peroxidase activity. The sections were then incubated with 5% normal horse serum or goat serum in TritonX-100/PBS overnight at 4°C with the following monoclonal primary antibodies: anti-Bax (Santa Cruz Biotech, SC-493, 1:100), anti-Bcl-xL (Transduction Laboratories, 610209, 1:100), anti-caspase-3 (Cell Signaling Technology, D175, 1:250), anti-BDNF (Chemicon, 1513P, 1:200), or anti-TrkB (Santa Cruz Biotech, sc-12, 1:250). After the sections were washed, they were incubated with a secondary antibody [biotinylated horse anti-mouse IgG (Jackson, 1:500) or biotinylated goat anti-rabbit IgG (Jacksbioton, 1:400)] for 2 h at room temperature. The sections were then incubated with avidinin-peroxidase complex (ABC; Vector, 1:200) for 2 h. The antigen-antibody reactions were visualized with 3, 3-diaminobenzidine 4-HCl (DAB, Sigma) or nickel-intensified DAB (to obtain better staining contrast). Throughout the experiment, PBS was used as the washing buffer after each step. All of the primary antibodies used in the present study have been validated specifically for use in avian species.

### Terminal deoxynucleotidyl transferase dUTP nick-end labeling (TUNEL) staining

The sections were incubated with 3% H_2_O_2_-methanol for 10 min at 15–25°C. The sections were then incubated in a freshly prepared 0.1% Triton X-100 solution containing 0.1% sodium citrate for 2 min on ice and in 50 μl of terminal deoxynucleotidyl transferase (TdT) dUTP nick-end labeling reaction solution for 60 min at 37°C according to the manufacturer’s instructions (TUNEL, Roche, Philadelphia, USA). These sections were protected from exposure to direct light during incubation.

### *In situ* hybridization

The primers for RNA probes were designed with the Primer3 output program (www-genome.wi.mit.edu/cgi-bin/primer/primer3-www.cgi), based on the sequences of the studied zebra finch genes published in GenBank of NCBI: bcl-2 (NM 205339.1) probe length: nucleotide (nt) 120 to 585, with sense primer from 120 to 139 and anti-sense primer from 566 to 585; bid (NM 204552.2) probe length: nt 262 to 623, with sense primer from 262 to 279 and anti-sense primer from 605 to 623; bad (NM 001285453.1) probe length: nt 93 to 485, with sense primer from 93 to 110 and anti-sense primer from 466 to 485. The total RNA was prepared from the brains of the P25 Bengalese finches using the TRIZOL reagent (GIBCO). Reverse transcription was performed with M-MLV (Promega). The resultant bands were cloned into pGEM-T Easy vector (Promega) and sequenced to confirm that they contained the desired sequence. The sense and anti-sense cRNA probes were transcribed according to the instructions of the manufacturer of the digoxigenin (DIG) RNA labeling kit (Roche). The corresponding sense probes were used as negative controls. These procedures have been detailed in a previous report [44].

### Electrical lesions of HVC and LMAN in juveniles

P18-22 birds were anesthetized via intramuscular injection of 20% barbiturate (30 μl/g body weight) and placed in a stereotaxic head holder. An electrode was inserted slowly into the target region based on the following coordinates: HVC: forward: 0.2–0.4 mm, left: 1.8–2.1 mm, depth: 0.3–0.5 mm; and LMAN: forward: 3.3–3.5 mm, right: 1.3–1.5 mm, depth: 3.3–3.5 mm. The electrical injuries (100 mA for 90 s) were induced unilaterally, with the other hemisphere kept intact as a control. After the lesions were made, each bird was returned to its original colony and kept with its family until P45.

### Neural tract tracing

The injured birds were anesthetized at P45 via intramuscular injection of 20% barbiturate (30 μl/g body weight) and placed in a stereotaxic head holder. Fluorogold was injected into the RA (backward: 1.1–1.4 mm; left/right: 2.2–2.3 mm; depth: 2.3–2.6 mm) of one cerebral hemisphere via a glass micropipette attached to a four-channel pressure injector (MDI, PM2000B). The birds were allowed to recover for four days and then were perfused and fixed with paraformaldehyde. The brains were then cut into 40 μm sections with a freezing microtome. The sections were incubated with anti-BDNF (Chemicon, 1513P, 1:200) primary antibody overnight at 4°C. After the sections were washed, they were incubated with an Alexa Fluor 488 Donkey Anti-Sheep IgG (H+L; Molecular Probe, A-11055, 1:40) secondary antibody for 2 h at room temperature.

### Photography, quantification of song nuclei size and number of labeled cells, and data analyses

Bright-field images of the targeted nuclei were captured with a color digital camera (Photometrics) attached to an Olympus microscope with QCapture Pro software. Fluorescent images were captured with an inverted fluorescence microscope (Axio Observer Z1, Zeiss) equipped with a monochromatic digital camera (AxioCam Mrm, Zeiss). The brightness and contrast of the images were modified with Adobe Photoshop CS5.

The Nissl-stained song control nuclei (HVC, RA, LMAN and Area X) and the labeled cells in the song nuclei were captured with ImageJ v.1.44 software (NIH Image program). The borders of the song nuclei were outlined, and the sizes were obtained with Image-Pro Plus 5.2. For the majority of the investigated song nuclei, the Nissl-defined borders could be clearly delineated from the surrounding tissues in both sexes at the ages studied (P15-120). However, the borders of the female LMAN after P45 and the female Area X at all of the studied ages were difficult to identify clearly. Thus, the approximate areas corresponding to the male song nuclei were determined with reference to the adjacent anatomic structures such as the lamina pallio-subpallialis (LPS). Similar to the male song nuclei, these areas in the female were generally characterized by the fact that they contain a greater number of large cells than the surrounding regions. The nuclei volumes were calculated by multiplying the sizes of the examined song nuclei by the section thickness. The densities of labeled cells were determined as the ratios of the total numbers of positive cells to the sizes of the examined areas. The total numbers of positive cells were determined as the densities of the labeled cells times the nuclei volumes. In immunohistochemistry, TUNEL and *in situ* hybridization studies, some of the borders of the song nuclei (particularly in the females) were determined with the aid of another set of Nissl-stained sections.

The statistical analyses were performed with SPSS 17.0 (SPSS Inc., Cary, NC, USA) and Prism 3.0 (GraphPad Software, San Diego, CA). We used two-way ANOVAs to examine the effects of gender and age and one-way ANOVAs examine between-sex differences at the same age. The distribution of each dependent variable was examined for normality prior to the application of an ANOVA, and the homogeneity of the variances was assessed for the equality of error variances (Levene’s test). Statistical significance was set at P<0.05.

## Results

### Volumetric changes in the Nissl-defined song control nuclei and caspase-3 expression in males and females

**Fig 1:**
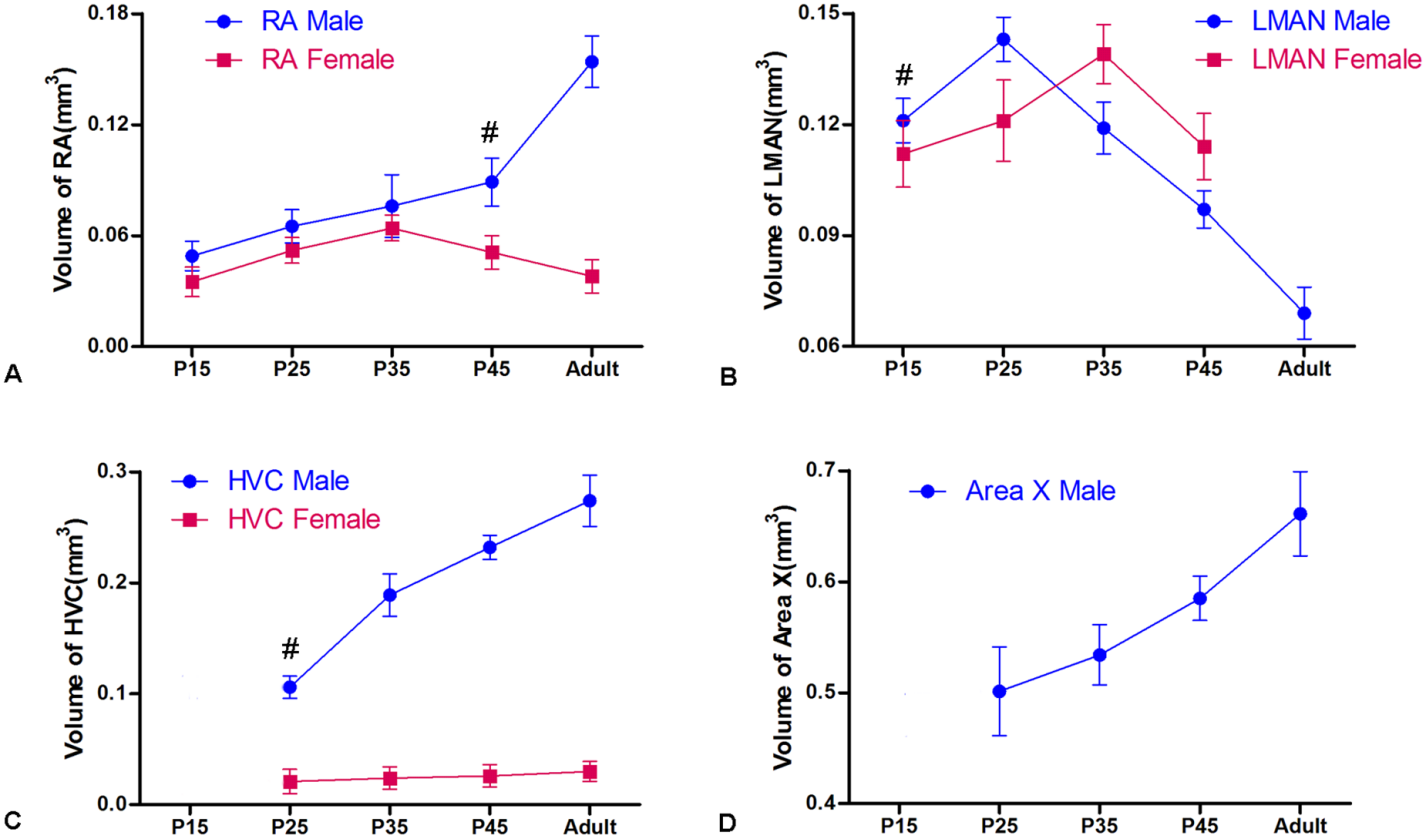
Nissl-defined volumetric changes of song control nuclei in the Bengalese finch from post-hatching day (P) 15 to 120. A: RA; B: LMAN; C: HVC; D: Area X; m: male; f: female. The mark “#” indicates that significant sexual differences are present in the marked groups towards adulthood. As the Nissl-defined borders of the female Area X in all studied age groups were difficult to clearly identify, the volume of the female Area X is not available in D.

As shown in Fig 1, RA was sexually dimorphic after P35 (t = 7.216, *P* = 0.003, n = 13, Fig 1A). There were large reductions in LMAN size among the males at P25 (*F*_(4, 35)_ = 19.604, *P* = 0.005, Fig 1B) and among the females at P35 (*F*_(3, 24)_ = 11.549, *P* = 0.003, Fig 1B). The sexual differences in HVC (*F*_(1, 64)_ = 56.304, *P* = 0.001, Fig 1C) and LAMN (*F*_(1, 56)_ = 33.314, *P* = 0.002, Fig 1B) across the examined age groups were significant. The Nissl-defined borders of the female Area X in all of the studied ages were difficult to identify clearly, so their volumes were not provided (Fig 1D).

**Fig 2:**
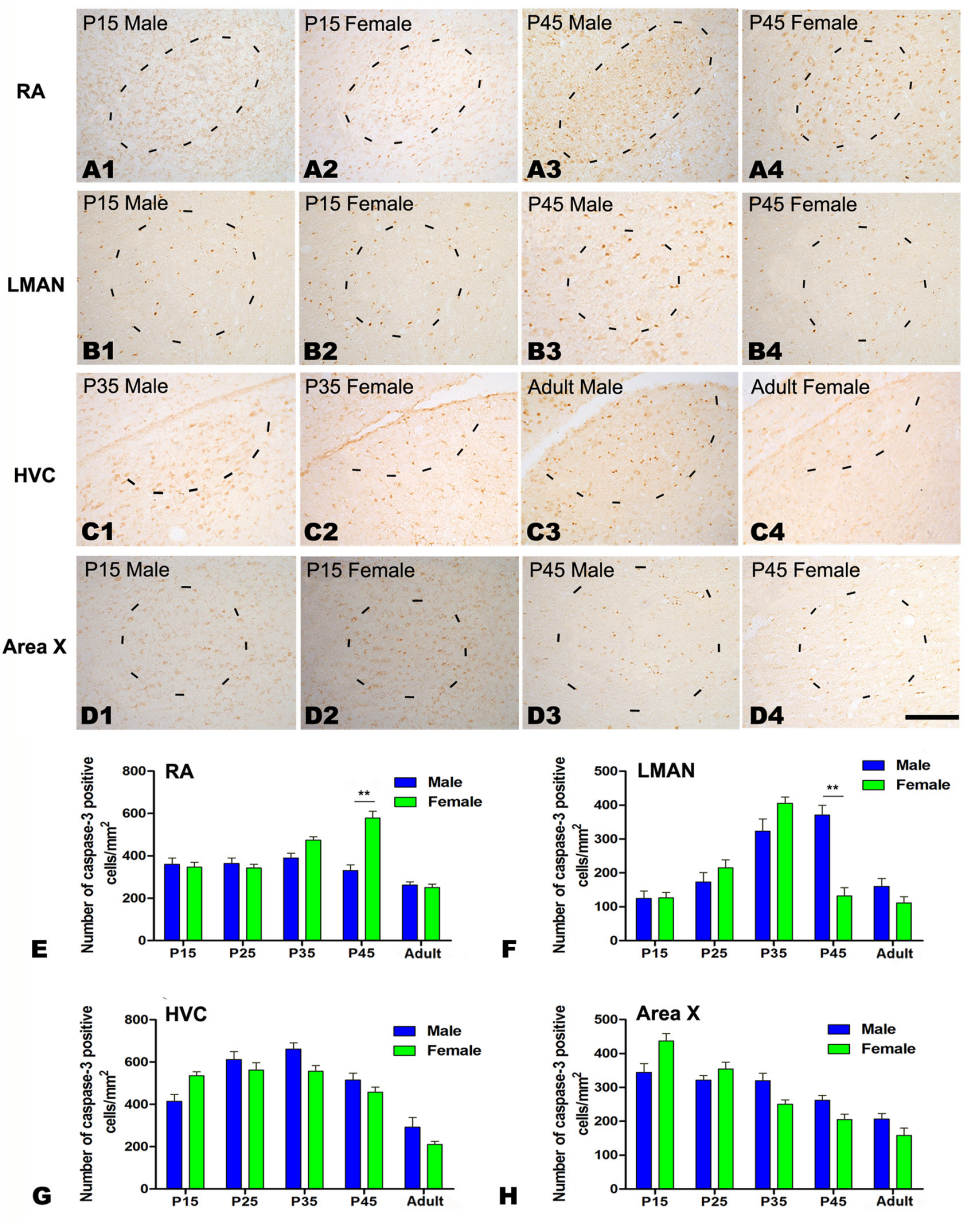
Immunohistochemistry for caspase-3 in the RA, LMAN, HVC and Area X in the Bengalese finch. A1–D4: Labeled cells are observed in the RA (A1–A4), LMAN (B1–B4), HVC (C1–C4) and Area X (D1–D4) in males at post-hatching days P15 (A1–D1) and P45 (A3–D3) and in females at P15 (A2–D2) and P45 (A4–D4). E-H: Comparison of the densities of caspase-3-positive cells in the RA (E), LMAN (F), HVC (G) and Area X (H) between males and females. Borders of the song nuclei (dashed lines) were determined with the help of another set of Nissl-stained sections. The Nissl-defined border of the female Area X was difficult to identify clearly, and the dashed lines in D2 and D4 indicate the approximate region that corresponds to the male Area X. Dorsal is up and caudal is right. Scale bar = 200 μm in A1–C4 and 300 μm in D1–D4. The data are expressed as the mean ± SEM. **P< 0.01.

A two-way ANOVA revealed that the number of caspase-3-positive cells per mm^2^ differed significantly among the five age groups in the RA (*F*_(4, 25)_ = 22.341, *P* <0.001, Fig 2A1–2A4 and 2E), LMAN (*F*_(4, 25)_ = 25.905, *P*<0.001, Fig 2B1–2B4 and 2F), HVC (*F*_(4, 25)_ = 40.271, *P*<0.001, Fig 2C1–2C4 and 2G) and Area X (*F*_(4, 25)_ = 34.821, *P*<0.001, Fig 2D1–2D4 and 2H). The differences between the sexes were also significant in the RA (*F*_(1, 25)_ = 14.664, *P* = 0.001, Fig 2A1–2A4 and 2E) and LMAN (*F*_(1, 25)_ = 20.331, *P*<0.001, Fig 2B1–2B4 and 2F), but not in the HVC (*F*_(1, 25)_ = 2.645, *P* = 0.12, Fig 2C1–2C4 and 2G) or Area X (*F*_(1, 25)_ = 1.33, *P* = 0.263, Fig 2D1–2D4 and 2H). Additional *t*-tests revealed further differences in the density of caspase-3-positive cells at P45 in the RA (t = –3.212, *P* = 0.004, n = 5, Fig 2E) and the LMAN (t = 6.289, *P* = 0.003, n = 5, Fig 2F), but these differences were not present in other age groups for the HVC and Area X (*F* values not shown, *P*>0.05, n = 5, Fig 2G and 2H).

### TUNEL staining in the four song control nuclei

**Fig 3:**
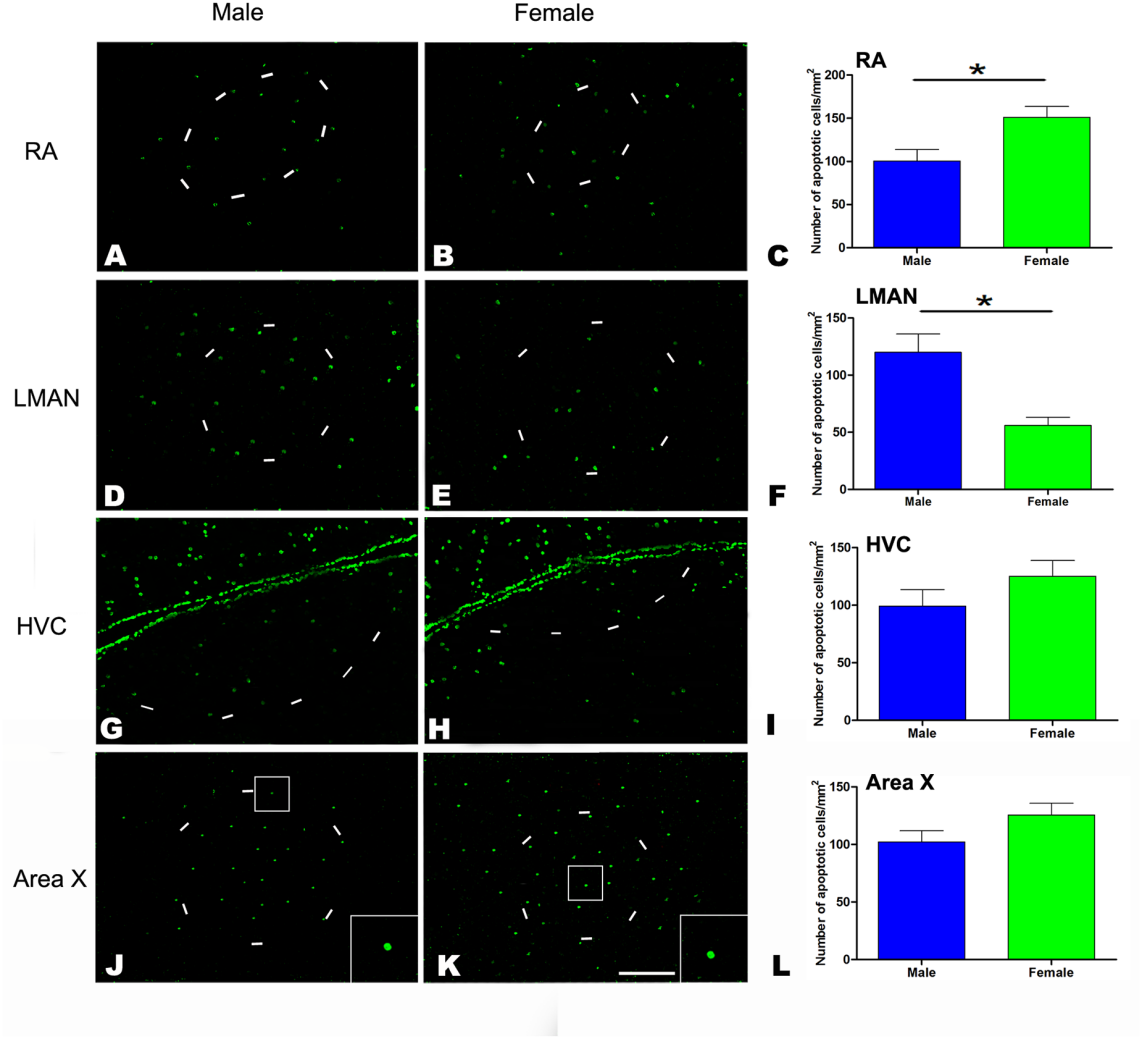
TUNEL labeling in the RA, LMAN, HVC and Area X of the Bengalese finch at post-hatching day (P) 45. A-K: Positive cells in RA (A, B), LMAN (D, E), HVC (G, H) and Area X (J, K). Comparison of the densities of TUNEL-positive cells between males and females in the RA (C), LMAN (F), HVC (I) and Area X (L). High magnification views of the labeled cells are shown in the insets (J, K). Scale bars = 400 μm in J and K and 200 μm in the other panels. The borders of the song nuclei (dashed lines) were determined with the help of another set of Nissl-stained sections. The Nissl-defined borders of the female Area X were difficult to clearly identify, and the dashed lines in K indicate the approximate region that corresponds to the male Area X. The data are expressed as the mean ± SEM. *P< 0.05.

To determine whether the extent of cell apoptosis revealed by caspase-3 immunochemistry was consistent with that revealed by TUNEL staining, we examined TUNEL labeling at P45 in the RA (Fig 3A and 3B), LMAN (Fig 3D and 3E), HVC (Fig 3G and 3H) and Area X (Fig 3J and 3K). Our results indicated that the number of TUNEL-positive cells was significantly different between the sexes at P45 in the RA (t = -2.89, *P* = 0.045, n = 4, Fig 3C) and LMAN (t = 3.627, *P* = 0.022, n = 4, Fig 3F), but not in the HVC (t = -2.19, *P* = 0.094, n = 4, Fig 3I) or Area X (t = -1.67, *P* = 0.17, n = 4, Fig 3L).

### Bax, Bcl-xL, BDNF and TrkB immunochemistry in the four song control nuclei

#### Bax immunochemistry

**Fig 4:**
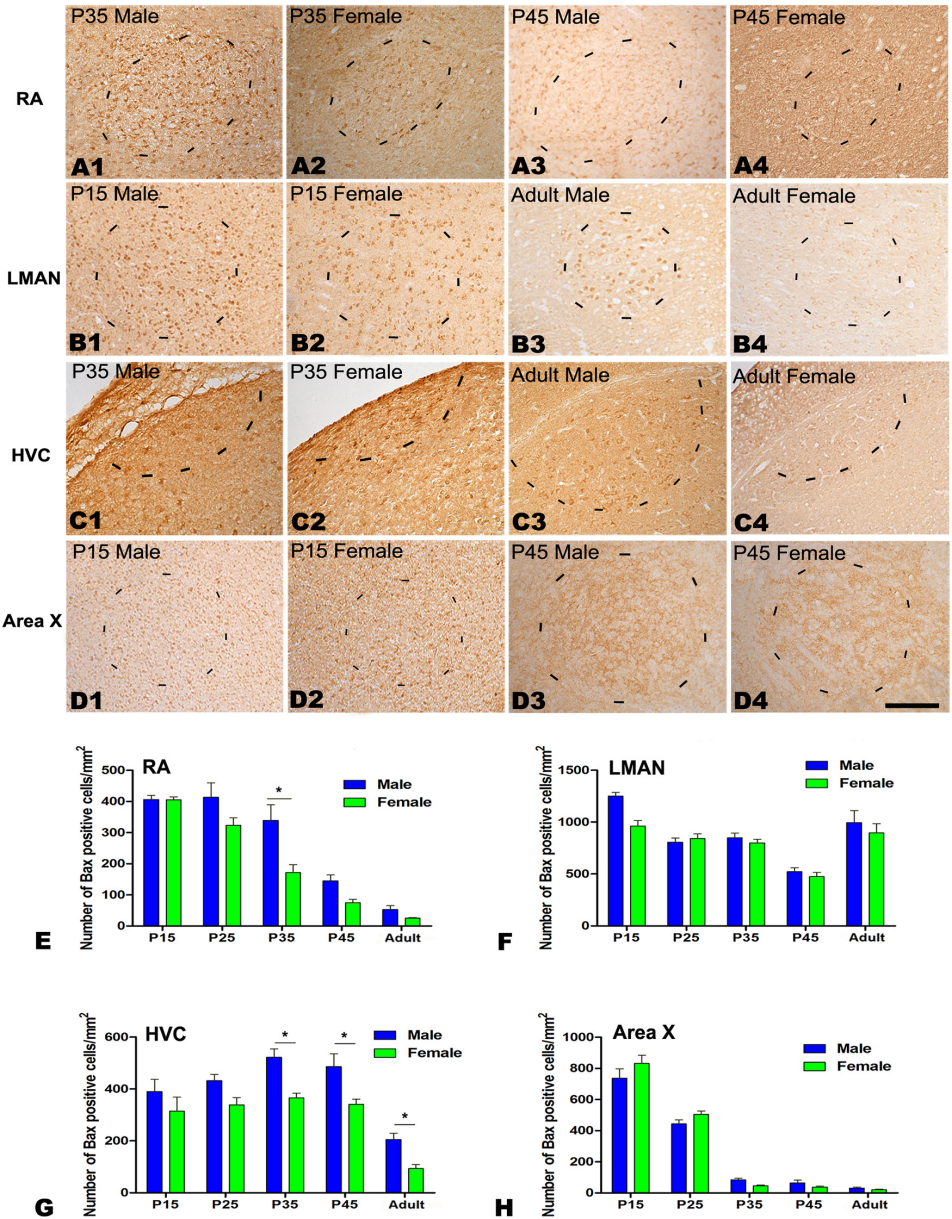
Immunohistochemistry for Bax in the RA, LMAN, HVC and Area X of the Bengalese finch. A1–D4: Labeled cells are observed in the RA at post-hatching day (P) 35 (A1, A2) and in the adult (A3–A4), in the LMAN at P15 (B1, B2) and in the adult (B3, B4), in the HVC at P35 (C1, C2) and in the adult (C3, C4), and in Area X at P15 (D1, D2) and P45 (D3, D4). E-H: Comparison of the densities of Bax-positive cells in the RA (E), LMAN (F), HVC (G) and Area X (H) between males and females. Borders of the song nuclei (dashed lines) were determined with the help of another set of Nissl-stained sections. The Nissl-defined border of the female Area X was difficult to clearly identify, and the dashed lines in D2 and D4 indicate the approximate region corresponding to the male Area X. Dorsal is up and caudal is right. Scale bar = 200 μm in A1–C4 and 300 μm in D1–D4. The data are expressed as the mean ± SEM. *P< 0.05.

As shown in Fig 4, the number of Bax-positive cells per mm^2^ differed significantly across the five age groups in the RA (*F*_(4, 24)_ = 74.027, *P*<0.001, Fig 4A1–4A4 and 4E), LMAN (*F*_(4, 24)_ = 35.913, *P*<0.001, Fig 4B1–4B4 and 4F), HVC (*F*_(4, 24)_ = 23.689, *P*<0.001, Fig 4C1–4C4 and 4G) and Area X (*F*_(4, 24)_ = 280.368, *P*<0.001 Fig 4D1–4D4 and 4H). Our results also revealed that the density of Bax-positive cells was significantly different between the sexes in the RA at P35 (t = 2.951, *P* = 0.042, n = 5, Fig 4A1–4A4 and 4E) and in the HVC after P35 (P35: t = 4.235, *P* = 0.013, n = 5; P45: t = 2.855, *P* = 0.046, n = 5; adult: t = 3.825, *P* = 0.019, n = 5, Fig 4C1–4C4 and 4G), but not in the LMAN or Area X in any of the studied groups.

#### Bcl-xL immunochemistry

**Fig 5:**
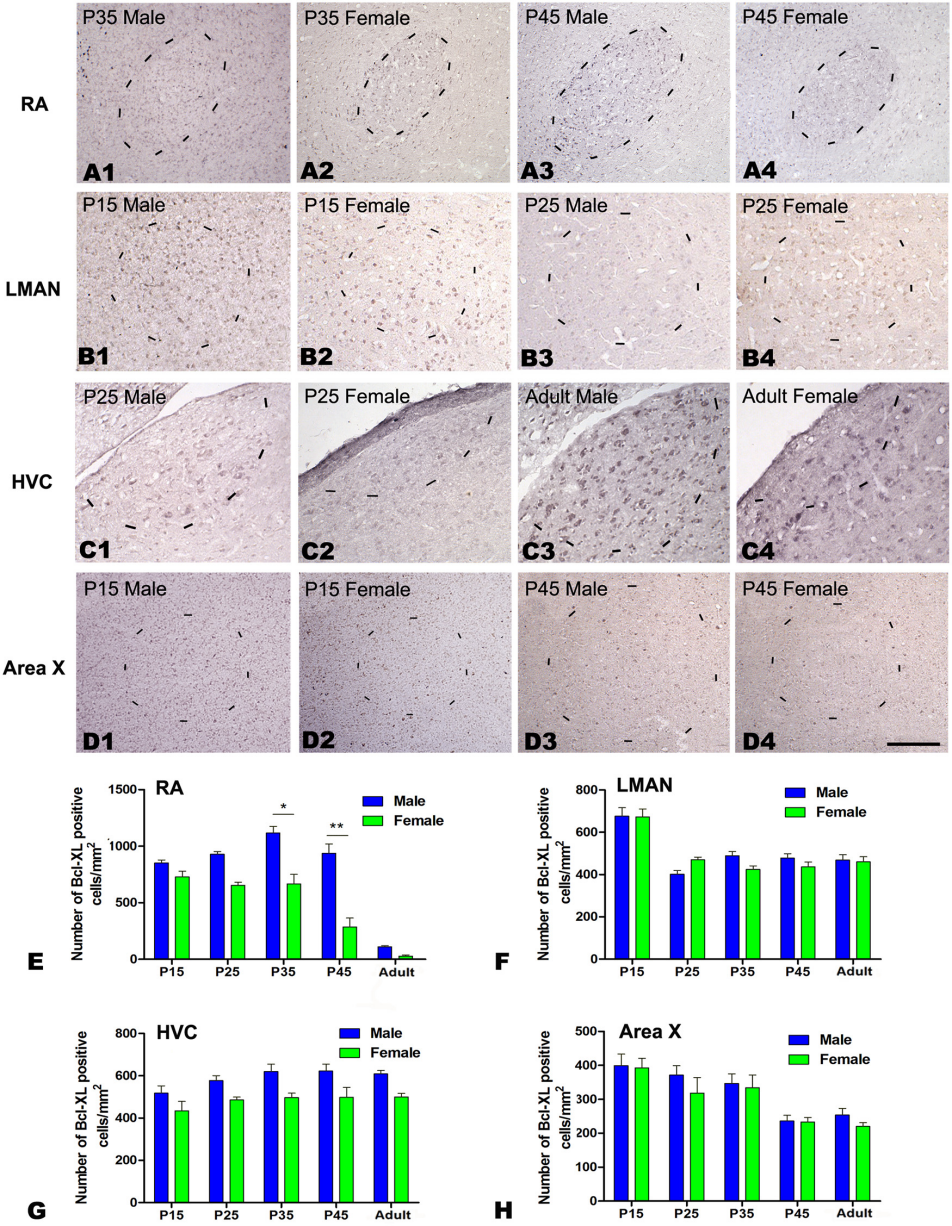
Immunohistochemistry for Bcl-xL in the RA, LMAN, HVC and Area X of the Bengalese finch (visualized by nickel intensified DAB). A1–D4: Labeled cells in the RA at post-hatching day (P) 35 (A1, A2) and P45 (A3–A4), in the LMAN at P15 (B1, B2) and P45 (B3, B4), in the HVC at P45 (C1, C2) and in the adult (C3, C4), and in Area X at P15 (D1, D2) and P45 (D3, D4). E-H: Comparison of the densities of Bcl-xL-positive cells in the RA (E), LMAN (F), HVC (G) and Area X (H) between males and females. The borders of the song nuclei (dashed lines) were determined with the help of another set of Nissl-stained sections. The Nissl-defined border of the female Area X was difficult to clearly identify, and the dashed lines in D2 and D4 indicate the approximate region corresponding to the male Area X. Dorsal is up and caudal is right. Scale bar = 200 μm in A1–C4 and 300 μm in D1–D4. The data are expressed as the mean ± SEM. *P< 0.05, **P< 0.01.

There were significant differences across the five age groups in the density of Bcl-xL-positive cells in the RA (Fig 5; *F*_(4, 22)_ = 77.600, *P*<0.001, Fig 5A1–5A4 and 5E), LMAN (*F*_(4, 22)_ = 31.171, *P*<0.001, Fig 5B1–5B4 and 5F) and Area X (*F*_(4, 22)_ = 8.218, *P*<0.001, Fig 5D1–5D4 and 5H), but not in HVC (*F*_(4, 22)_ = 2.639, *P* = 0.064, Fig 4C1–4C4 and 4G). The densities of Bcl-xL-positive cells were significantly different between the sexes in the RA at P35 (t = 4.394, *P* = 0.012, n = 4) and P45 (t = 5.721, *P* = 0.005, n = 4, Fig 5A1–5A4 and 5E), but not in the LMAN (*F*_(1, 22)_ = 0.394, *P* = 0.537, Fig 5B1–5B4 and 5F), HVC (*F*_(1, 22)_ = 1,439, *P* = 0.172, Fig 5C1–5C4 and 5G) or Area X (*F*_(1, 22)_ = 3.057, *P* = 0.096, Fig 5D1–5D4 and 5H) in any of the studied age groups.

#### BDNF and TrkB immunochemistry

**Fig 6:**
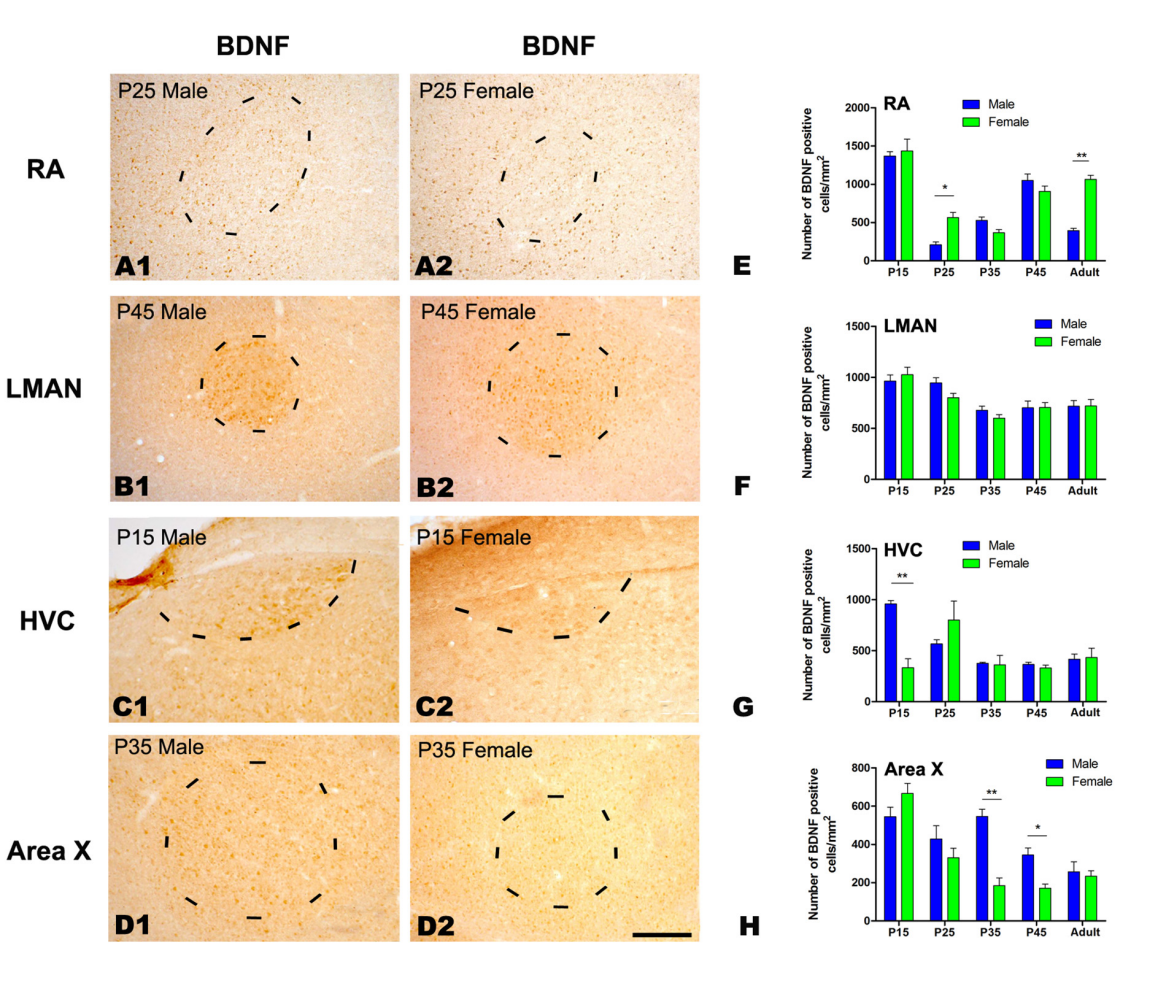
Immunohistochemistry for BDNF in the RA, LMAN, HVC and Area X of the Bengalese finch. A1–D2: Labeled cells in the RA at post-hatching day (P) 25 (A1, A2), the LMAN at P45 (B1, B2), the HVC at P15 (C1, C2) and in Area X at P35 (D1, D2). E-H: Comparisons of the densities of BDNF-positive cells in the RA (E), LMAN (F), HVC (G) and Area X (H) between males and females. Borders of the song nuclei (the dashed lines) were determined with the help of another set of Nissl-stained sections. The Nissl-defined border of the female Area X was difficult to clearly identify, and the dashed lines in D2 indicate the approximate region corresponding to the male Area X. Dorsal is up and caudal is right. Scale bar = 200 μm in A1–C2 and 300 μm in D1–D2. The data are expressed as the mean ± SEM. *P< 0.05, **P< 0.01.

**Fig 7:**
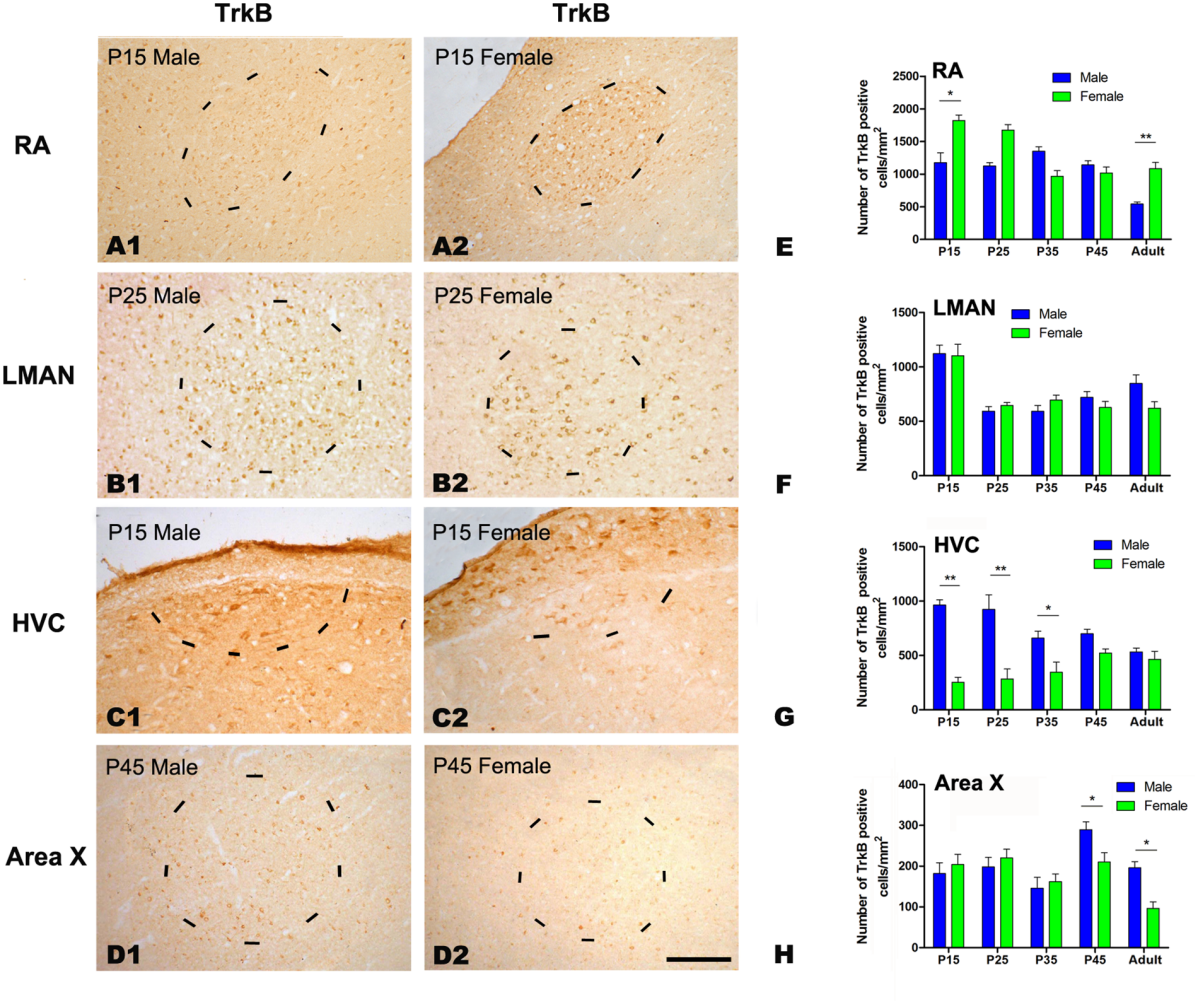
Immunohistochemistry for TrkB in the RA, LMAN, HVC and Area X in the Bengalese finch. A1–D2: Labeled cells in the RA at post-hatching day (P) 15 (A1, A2), the LMAN at P25 (B1, B2), the HVC at P15 (C1, C2), and in Area X at P45 (D1, D2). E-H: Comparisons of the densities of TrkB-positive cells in the RA (E), LMAN (F), HVC (G) and Area X (H) between males and females. Borders of the song nuclei (dashed lines) were determined with the help of another set of Nissl-stained sections. The Nissl-defined border of the female Area X was difficult to clearly identify, and the dashed lines in D2 indicate the approximate region corresponding to the male Area X. Dorsal is up and caudal is right. Scale bar = 200 μm in A1–C2 and 300 μm in D1–D2. The data are expressed as the mean ± SEM. *P< 0.05, **P< 0.01.

We compared the distributions of BDNF- and TrkB-positive cells in the four studied song nuclei of both sexes (BDNF: Fig 6; TrkB: Fig 7). Our results indicated that the densities of BDNF- and TrkB-positive cells were significantly different across the five age groups in the RA (BDNF: *F*_(4, 23)_ = 66.667, *P*<0.001, Fig 6A1, 6A2 and 6E; TrkB: *F*_(4, 23)_ = 20.035, *P*<0.001, Fig 7A1, 7A2 and 7E), LMAN (BDNF: *F*_(4, 23)_ = 13.763, *P*<0.001, Fig 6B1, 6B2 and 6F; TrkB: *F*_(4, 23)_ = 20.603, *P*<0.001, Fig 7B1, 7B2 and 7F), HVC (BDNF: *F*_(4, 23)_ = 7.662, *P* = 0.001, Fig 6C1, 6C2 and 6G) and Area X (BDNF: *F*_(4, 23)_ = 20.328, *P*<0.001, Fig 6D1, 6D2 and 6H; TrkB: *F*_(4, 23)_ = 7.603, *P* = 0.001, Fig 7D1, 7D2 and 7H). However, TrkB density did not differ in the HVC (TrkB: *F*_(4, 23)_ = 1.325, *P* = 0.295, Fig 7C1, 7C2 and 7G). There were significant sexual differences in the density of BDNF-positive cells in the RA at P25 (t = -4.610, *P* = 0.001, n = 5, Fig 6A1, 6A2 and 6E) and in the adult (t = -11.156, *P*<0.001, n = 5, Fig 6E), in the HVC at P15 (t = 6.705, *P* = 0.003, n = 4, Fig 6C1, 6C2 and 6G), and in the Area X at P35 (t = 6.626, *P* = 0.003, n = 5, Fig 6D1, 6D2 and 6H) and P45 (t = 4.114, *P* = 0.015, n = 5, Fig 6H), but no differences were found in the LMAN (*F*_(1, 23)_ = 0.788, *P* = 0.385, Fig 6B1, 6B2 and 6F). Similar to BDNF, there were significant sexual differences in the density of TrkB-positive cells in the RA at P15 (t = -3.700, *P* = 0.021, n = 5, Fig 7A1, 7A2 and 7E) and in the adult (t = -5.434, *P* = 0.006, n = 5, Fig 7E), in the HVC at P15 (t = 10.920, *P*<0.001, n = 4, Fig 7C1, 7C2 and 7G), P25 (t = 12.825, *P*<0.001, n = 5, Fig 7G) and P35 (t = 2.825, *P* = 0.048, n = 5, Fig 7G), and in the Area X at P45 (t = 3.708, *P* = 0.049, n = 5, Fig 7D1, 7D2 and 7H) and in the adult (t = 4.511, *P* = 0.011, n = 5, Fig 7H), but no differences were present in the LMAN for any of the studied age groups (*F*_(1, 23)_ = 0.862, *P* = 0.346, Fig 7B1, 7B2 and 7F).

### *In situ* hybridization for Bcl-2, Bid and Bad

**Fig 8:**
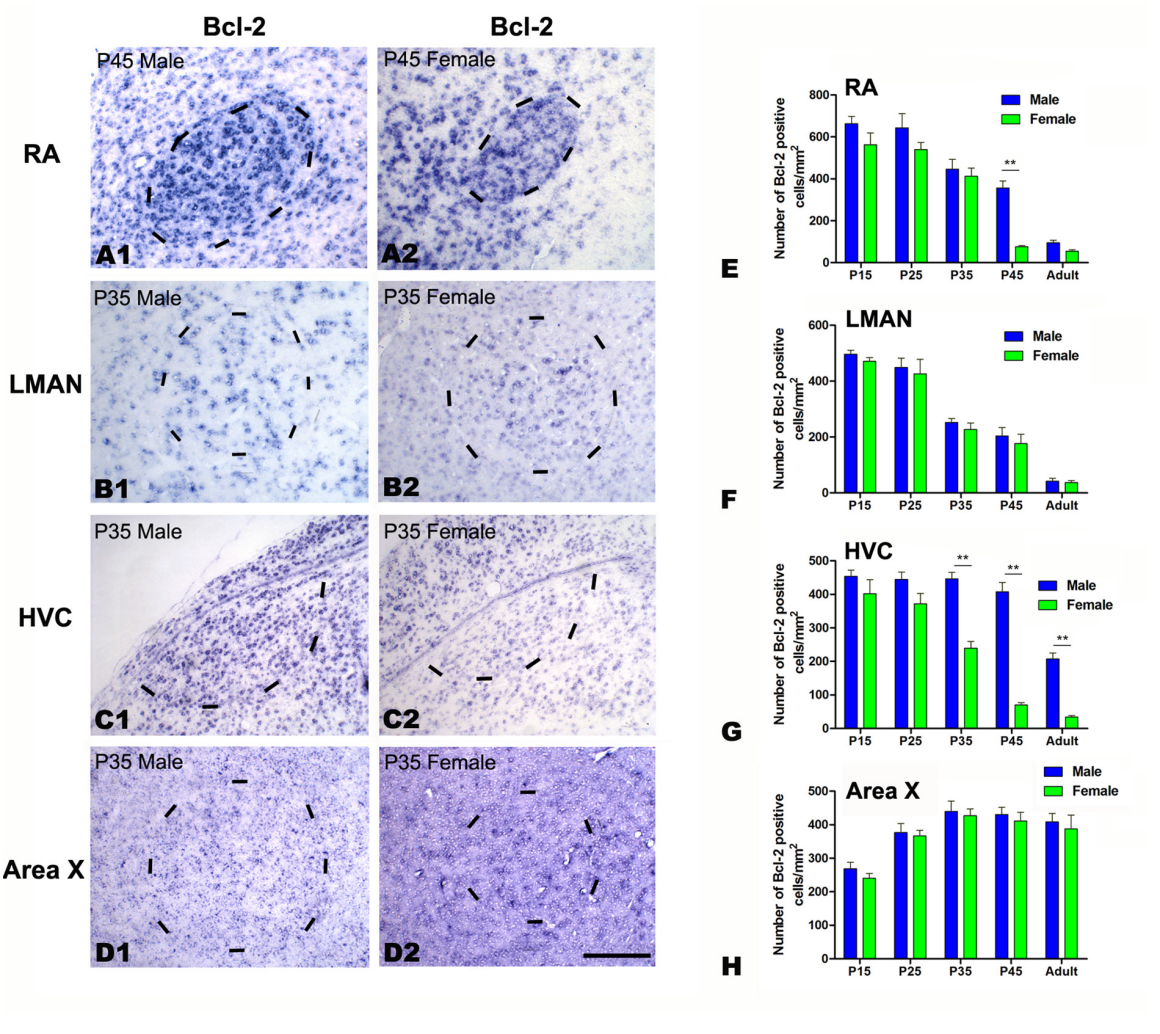
*In situ* hybridization for Bcl-2 mRNA in the RA, LMAN, HVC and Area X in the Bengalese finch. A1–D2: Labeled cells in the RA at post-hatching day (P) 45 (A1, A2), the LMAN at P35 (B1, B2), the HVC at P35 (C1, C2), and in Area X at P35 (D1, D2). E-H: Comparisons of the densities of Bcl-2 mRNA-positive cells in the RA (E), LMAN (F), HVC (G) and Area X (H) between males and females. Borders of the song nuclei (dashed lines) were determined with the help of another set of Nissl-stained sections. The Nissl-defined border of the female Area X was difficult to clearly identify, and the dashed lines in D2 indicate the approximate region corresponding to the male Area X. Dorsal is up and caudal is right. Scale bar = 200 μm in A1–C2 and 300 μm in D1–D2. The data are expressed as the mean ± SEM. **P< 0.01.

**Fig 9:**
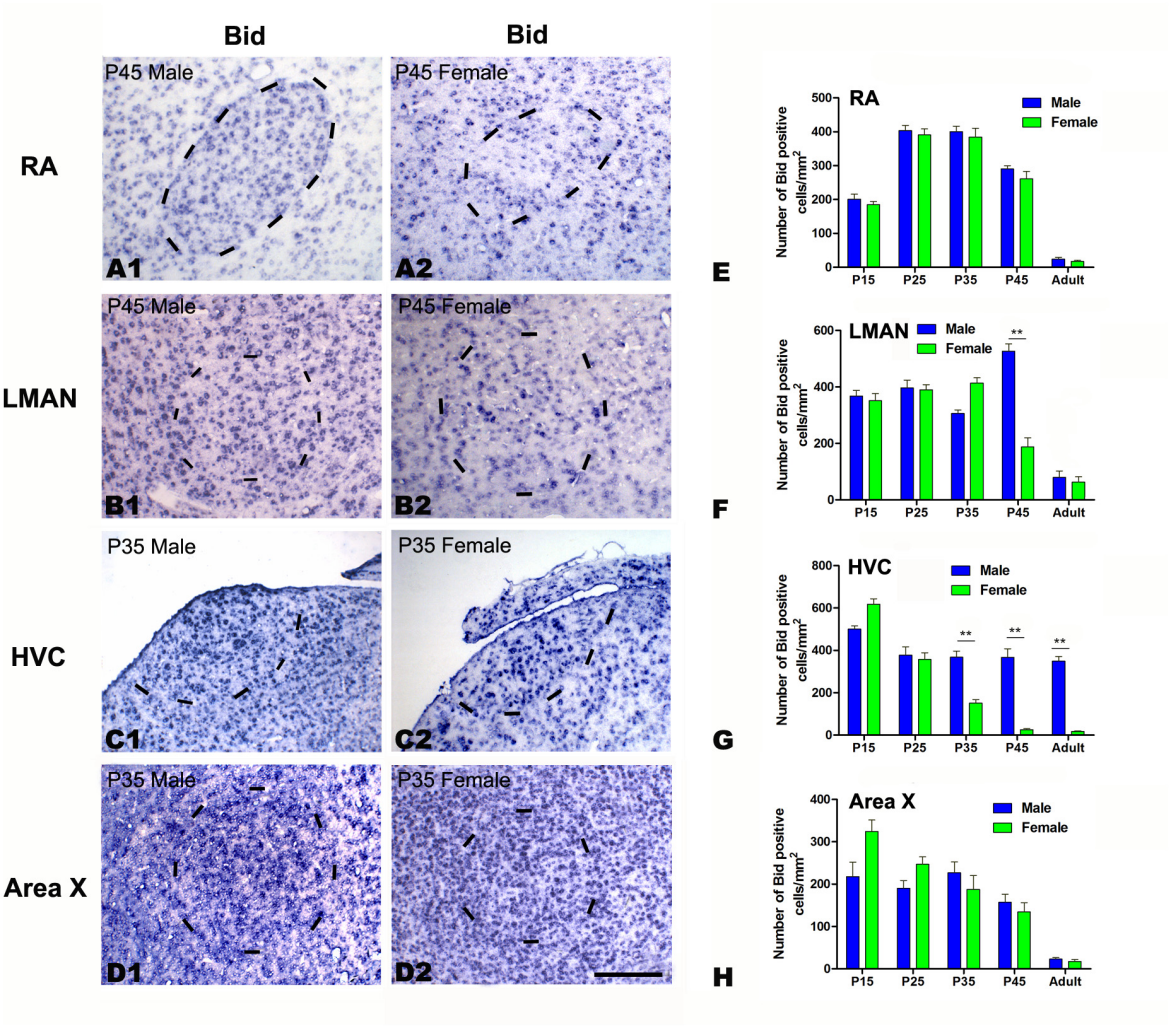
*In situ* hybridization for Bid mRNA in the RA, LMAN, HVC and Area X of the Bengalese finch. A1–D2: Labeled cells in the RA at post-hatching day (P) 45 (A1, A2), the LMAN at P45 (B1, B2), the HVC at P35 (C1, C2), and in Area X at P15 (D1, D2). E-H: Comparisons of the densities of Bid mRNA-positive cells in the RA (E), LMAN (F), HVC (G) and Area X (H) between males and females. Borders of the song nuclei (dashed lines) were determined with the help of another set of Nissl-stained sections. The Nissl-defined border of the female Area X was difficult to clearly identify, and the dashed lines in D2 indicate the approximate region corresponding to the male Area X. Dorsal is up and caudal is right. Scale bar = 200 μm in A1–C2 and 300 μm in D1–D2. The data are expressed as the mean ± SEM. **P< 0.01.

**Fig 10:**
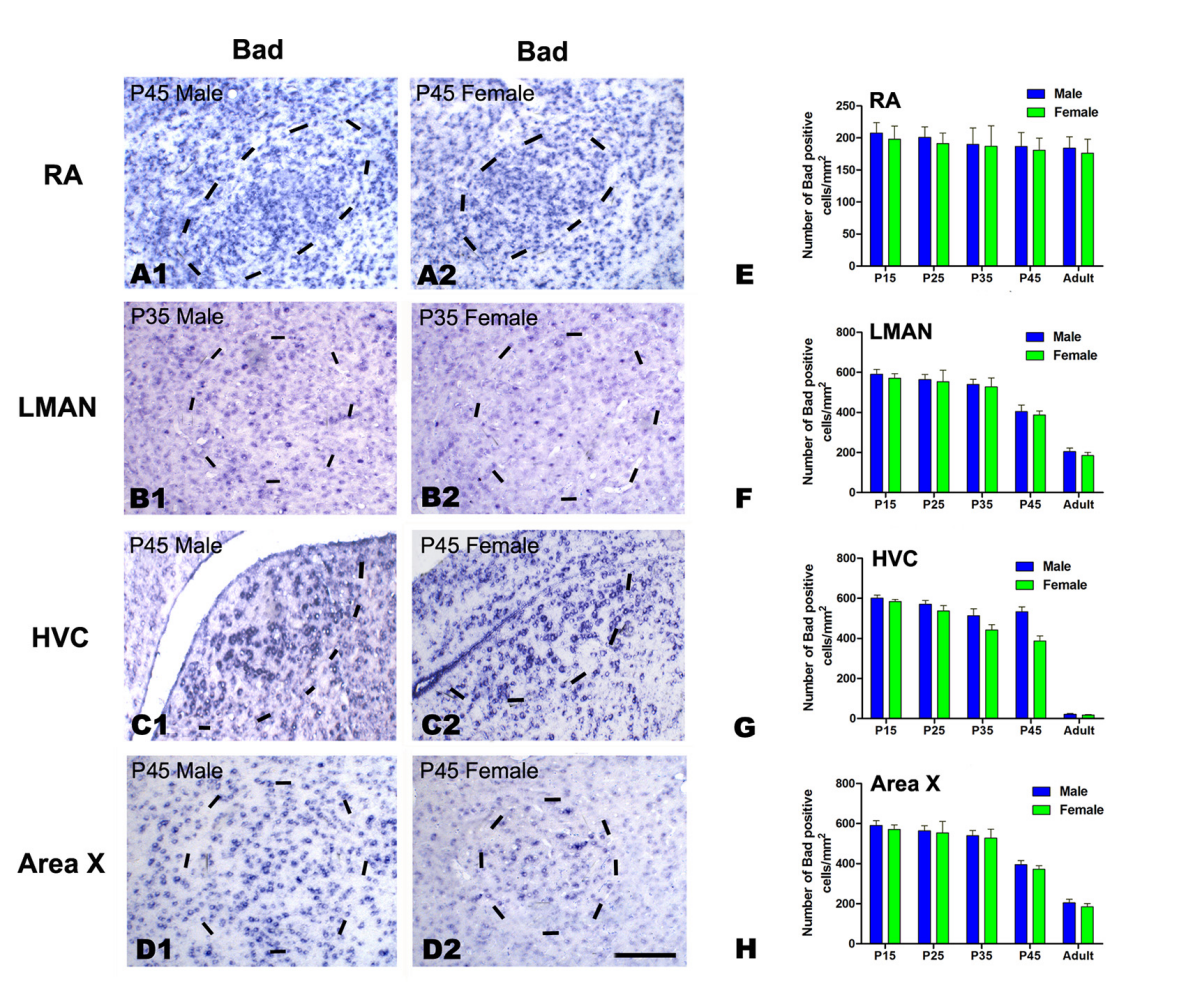
*In situ* hybridization for Bad mRNA in the RA, LMAN, HVC and Area X of the Bengalese finch. A1–D2: Labeled cells in the RA at post-hatching day (P) 45 (A1, A2), the LMAN at P35 (B1, B2), the HVC at P45 (C1, C2), and in Area X at P35 (D1, D2). E-H: Comparisons of the densities of Bad mRNA-positive cells in the RA (E), LMAN (F), HVC (G) and Area X (H) between males and females. Borders of the song nuclei (dashed lines) were determined with the help of another set of Nissl-stained sections. The Nissl-defined border of the female Area X was difficult to clearly identify, and the dashed lines in D2 indicate the approximate region corresponding to the male Area X. Dorsal is up and caudal is right. Scale bar = 200 μm in A1–C2 and 300 μm in D1–D2. The data are expressed as the mean ± SEM.

As commercial antibodies for Bcl-2, Bid and Bad are not available for avian species, we examined the mRNA expression of these genes in the four song control nuclei at P15, 25, 35, and 45 and in the adult (Bcl-2: Fig 8; Bid: Fig 9; Bad: Fig 10). The densities of Bcl-2, Bid and Bad mRNA-positive cells exhibited significant differences across the five age groups in nearly all of the studied song nuclei, including the RA (Bcl-2: *F*_(4, 20)_ = 73.630, *P*<0.001, Fig 8A1, 8A2 and 8E; Bid: *F*_(4, 20)_ = 202.123, *P*<0.001, Fig 9A1, 9A2 and 9E), LMAN (Bcl-2: *F*_(4, 20)_ = 94.570, *P*<0.001, Fig 8B1, 8B2 and 8F; Bid: *F*_(1, 20)_ = 74.784, *P*<0.001, Fig 9B1, 9B2 and 9F; Bad: *F*_(4, 20)_ = 54.582, *P*<0.001, Fig 10B1, 10B2 and 10F), HVC (Bcl-2: *F*_(4, 20)_ = 61.155, *P*<0.001, Fig 8C1, 8C2 and 8G; Bid: *F*_(4, 20)_ = 71.859, *P*<0.001, Fig 9C1, 9C2 and 9G; Bad: *F*_(4, 20)_ = 237.056, *P*<0.001, Fig 10C1, 10C2 and 10G), and Area X (Bcl-2: *F*_(4, 20)_ = 15.974, *P*<0.001, Fig 8D1, 8D2 and 8H; Bid: *F*_(1, 20)_ = 1.777, *P* = 0.197, Fig 9D1, 9D2 and 9H; Bad: *F*_(4, 20)_ = 59.621, *P*<0.001, Fig 10D1, 10D2 and 10H). However, the densities of Bad-positive cells did not differ significantly across the five age groups in the RA (*F*_(4, 20)_ = 0.374, *P* = 0.824, Fig 10A1, 10A2 and 10E). There were significant sex differences in the densities of Bcl-2-positive cells in the RA at P45 (t = 8.264, *P* = 0.001, n = 4, Fig 8A1, 8A2 and 8E) and in the HVC at P35 (t = 7.750, *P* = 0.001, n = 4, Fig 8C1, 8C2 and 8G), P45 (t = 11.664, *P*<0.001, n = 4, Fig 8G) and in the adult (t = 9.421, *P* = 0.001, n = 4, Fig 8G). However, no differences were found in any of the studied age groups in the LMAN (*F*_(1, 20)_ = 1.588, *P* = 0.222, Fig 8B1, 8B2 and 8F) or Area X (*F*_(1, 20)_ = 3.125, *P* = 0.452, Fig 8D1, 8D2 and 8H). The density of Bid-positive cells differed significantly between the sexes in the LMAN at P45 (t = 8.181, *P* = 0.001, n = 4, Fig 9A1, 9A2 and 9E) and in the HVC at P35 (t = 6.462, *P* = 0.003, n = 4, Fig 9C1, 9C2 and 9G), P45 (t = 8.344, *P* = 0.001, n = 4, Fig 9G) and in the adult (t = 15.534, *P*<0.001, n = 4, Fig 9G), but were not sexually dimorphic in any of the studied age groups in the RA (*F*_(1, 20)_ = 2.717, *P* = 0.155, Fig 9E) or Area X (*F*_(1, 20)_ = 1.777, *P* = 0.197, Fig 9H). Additionally, there was not a significant sex difference in Bad-positive cell density in any of the studied age groups in the HVC (*F*_(1, 20)_ = 16.230, *P* = 0.001, Fig 10C1, 10C2 and 10G), RA (*F*_(1, 20)_ = 0.288, *P* = 0.597 Fig 10A1, 10A2 and 10E), LMAN (*F*_(1, 20)_ = 0.642, *P* = 0.432 Fig 10B1, 10B2 and 10F) or Area X (*F*_(1, 20)_ = 0.815, *P* = 0.377, Fig 10D1, 10D2 and 10H).

### LMAN and HVC lesions and neural tract tracing in juvenile Bengalese finches

**Fig 11:**
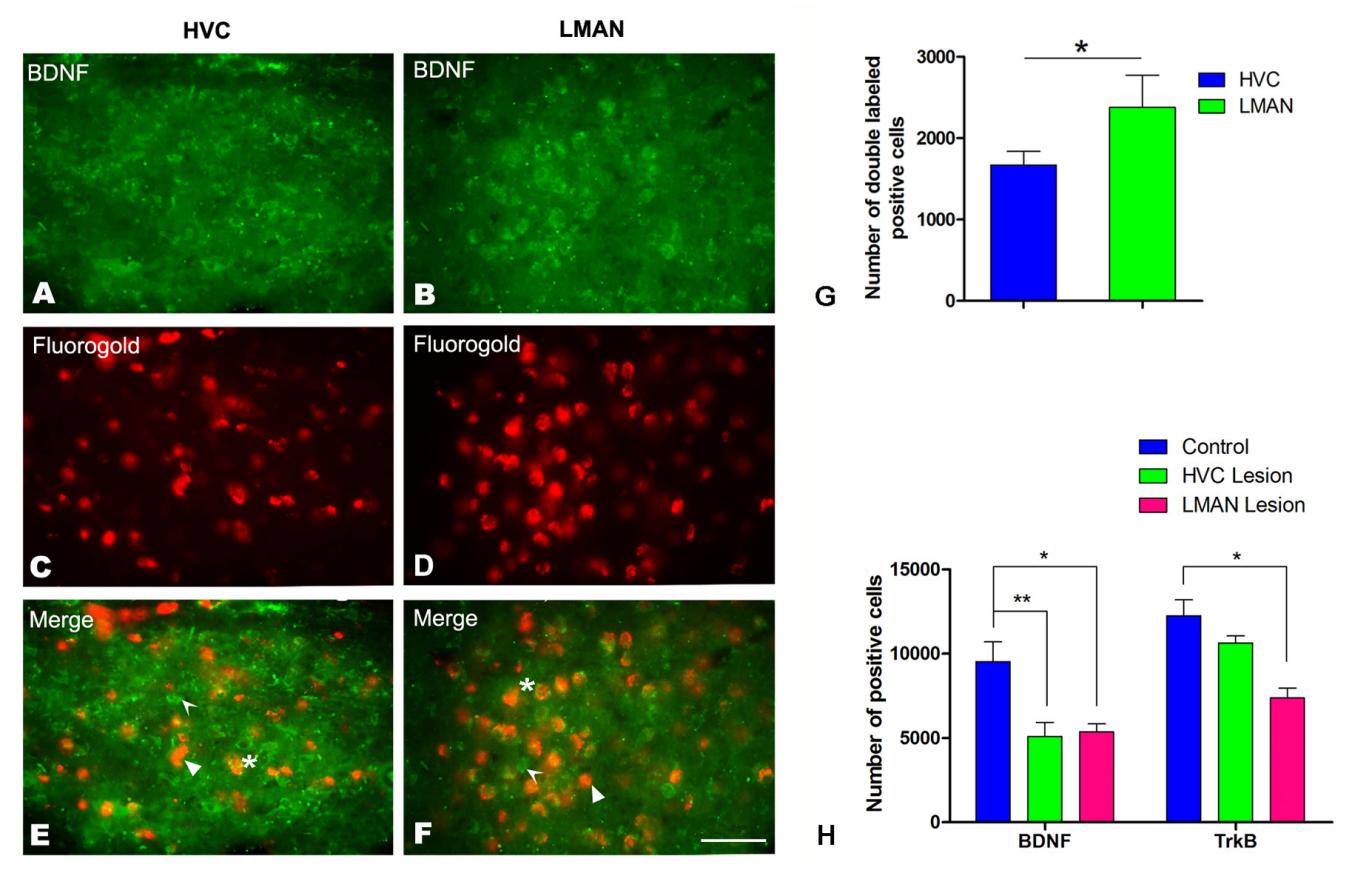
BDNF immunoreactive cells and retrogradely labeled cells in the HVC and LMAN at post-hatching day (P) 45 after injection of fluorogold into the RA. A and B: BDNF-labeled positive cells in the HVC (A) and LMAN (B). C and D: Retrogradely labeled cells in the HVC (C) and LMAN (D) after injection of fluorogold into the RA. E and F: Merged images of A and C (E) and of B and D (F). The arrows in E and F indicate BDNF-positive cells, the curved arrows indicate fluorogold-positive cells, and the asterisks indicate double-labeled cells. Scale bar = 200 μm. G: The total numbers of BDNF- or TrkB-positive cells in the RA after unilateral HVC or LMAN lesion at P45. H: The total number of BDNF-positive cells projecting to the RA at P45 in the HVC or LMAN. The data are expressed as the mean ± SEM. *P< 0.05.

We next compared the changes in the number of BDNF/TrkB-positive cells in the male RA at P45, following the electrical lesion of its two upstream nuclei, the LMAN and HVC, in one hemisphere at P18–22. The total numbers of BDNF- or TrkB-positive cells in the RA following LMAN or HVC lesion compared to the RA of the intact hemisphere are shown in Fig 11G. After LMAN lesion, RA volumes decreased by 58.3±8.1% compared to the RA in the intact hemisphere (t = 7.265, *P* = 0.002, n = 12), and the total numbers of BDNF- or TrkB-positive cells decreased by 48.9±5.5% (t = 4.623, *P* = 0.010, n = 10) and 12.5±1.3% (t = 4.372, *P* = 0.012, n = 10), respectively, compared to the intact hemisphere (Fig 11G). Following HVC lesion, RA volume decreased by 28.4% ± 5.6% (t = 5.866, *P* = 0.001, n = 10), compared to the intact hemisphere, and the total numbers of BDNF- or TrkB-positive cells in the RA decreased by 59.3% ± 6.8% (t = 3.076, *P* = 0.007, n = 10) and 53.3±7.2% (t = 6.511, *P* = 0.001, n = 10), respectively, compared to the intact hemispheres.

To further determine whether the cells that project from the LMAN or HVC transport BDNF into the RA, we examined and compared the total number of cells that were double-labeled for fluorogold (which marked cells projecting to the RA) and BDNF in relation to the total number of BDNF-positive cells in the HVC and LMAN after the injection of fluorogold into the male RA at P45. The total number of BDNF-positive cells projecting to the RA from the HVC (Fig 11A, 11C and 11E) or from the LMAN (Fig 11B, 11D and 11F) (n = 5) is shown in Fig 11H (the values were obtained by multiplying the density of the cells that were double-labeled for fluorogold and BDNF in the LMAN or HVC by the volume of the nucleus). Our results further indicated that the percentage of BDNF-positive cells double-labeled by fluorogold was 38.1±4.1% in the LMAN and 19.8±3.4% in the HVC, and the total numbers of double-labeled cells in the HVC and LMAN were 1670.2±167.6 and 2379.6±395.3, respectively (Fig 11H). Both the percentages and the total numbers revealed significant differences between the HVC and LMAN (t = 11.147, *P* = 0.005, n = 5, Fig 11H).

## Discussion

### Comparison with previous studies

Following the pioneering report by Nottebohm and Arnold [2], sexual differences in song control nuclei have been identified in many other oscine species, although most reports have focused on the zebra finch. As shown in the present study, the size of the RA in the Bengalese finch exhibited significant differences after P35, and the sizes of both the HVC and LMAN exhibited significant differences at all ages examined. The outlines of the female Area X at all examined ages and the female LMAN after P45 could not be clearly identified. Despite this ambiguity, these areas (tentatively identified by their correspondence to the respective male Area X or LMAN) were characterized by virtue of containing more Nissl-stained cells of medium and large size compared to the surrounding regions. These results are similar to those reported for the zebra finch, which is consistent with the fact that the Bengalese finch and the zebra finch belong to the same family (Estrildidae) [14, 45–47].

According to previous reports, cells produced in the ventricular zone migrate to the HVC and Area X before the critical period (approximately 30 days after hatching), while cells in the RA and LMAN are produced prior to hatching [9–12]. Due to neuronal death in the female RA after P30 and a loss of more than 50% of the neurons in both the male and female LMAN, nucleus sizes decrease markedly in the zebra finch [10, 11, 48–50]. The present study showed that the densities of caspase-3-expressing cells were much higher in the female RA at P45 and in both the male and female LMAN during the period of cell loss (P35 in the female LAMN and P45 in the male LMAN) than at any of the earlier stages. Our study further showed that the number of TUNEL-labeled cells differed between the sexes at P45, within the period of cell loss in the aforementioned song nuclei. Consistent with the observation that the size of the LMAN decreased more rapidly in males than in females after P35, the densities of both caspase-3- and TUNEL-labeled cells were much higher in males than in females. Considering that caspase-3 can trigger neuronal cell death by proteolyzing endonucleases that lead to DNA cleavage [51, 52] and that caspase-3 has been used as a tool to detect cell apoptosis *in vivo* [31, 53], our data suggest that the obvious loss of cells in the female RA and in the male and female LMAN were due to apoptosis mediated by caspase-3. However, we did not observe significant differences in the densities of caspase-3- or TUNEL-labeled cells in the HVC or Area X, consistent with a report that the densities and number of TUNEL-labeled or pyknotic cells visible in Nissl-stained sections within the HVC and its overlying ventricular zone do not differ between normal 20- and 30-day-old male and female zebra finches [37]. Quantification of the labeled cells was presented only as the density (the number of labeled cells/the area of the examined song nuclei) and not as the total number of labeled cells in a nucleus for two reasons. First, the boundaries of the female Area X and female LMAN after P45 could not be clearly identified; thus, the total number of labeled cells in these song nuclei could not be obtained precluding comparisons between the two sexes. Second, the two measures (i.e., the density and the total number of labeled cells in song nuclei) are highly correlated [9, 10]; therefore, we presented only one.

### Mechanism of apoptosis in song control nuclei

The molecular mechanisms of apoptosis are well characterized. Both Bcl-2 and Bcl-xL are important anti-apoptotic proteins, and any deficiency or over-expression of these proteins can cause extensive neuronal death or a decrease in neuronal apoptosis and subsequent increase in neuron number [54]. Although the over-expression of Bcl-2 can decrease neuronal apoptosis and increase neuron number in some brain regions [54], Bcl-2 disruption results in only subtle neural abnormalities and an increase in neuronal apoptosis, which suggests that other apoptotic factors are required [55, 56]. In addition to a report showing that pro-apoptotic proteins (including Bax, Bid and Bad) are involved in cell apoptosis [57], some studies have indicated that the ratio of anti-apoptotic to pro-apoptotic proteins (e.g., Bcl-xL or Bcl-2/Bax) can determine cell fate following an apoptotic stimulus, and decreases in such ratios are accompanied by apoptosis, while increases in the ratio have the opposite effect [21, 58, 59].

As shown above, the size of the female RA decreased markedly due to neuronal apoptosis at P45. In respective age groups, there were significant differences in the densities of Bcl-xL and Bcl-2 between males and females (male>female), but there were not significant differences in the densities of pro-apoptotic members. These data suggest that the high expression levels of two anti-apoptotic members (Bcl-xL or Bcl-2) might protect the male, but not female, RA from cell apoptosis, resulting in sexual dimorphism in the RA. In the LMAN, there were no significant differences in the densities of Bcl-xL, Bcl-2 or the other pro-apoptotic members, with the exception of Bid at P45 (male>female). It should be noted that there was a sharp decrease in the density of Bcl-2 in the LMAN after P35 (greater than 100% compared to the younger age groups, P15 and P25). As shown above, although the size of both the male and female LMAN decreased markedly after P35, it decreased more rapidly in males than in females. Accompanying the changes in LMAN size, none of the pro-apoptotic or anti-apoptotic members, except for Bid, differed significantly between the sexes, suggesting that Bid is probably involved in apoptosis to a greater extent in the male LMAN. Similarly, our results show that no pro- or anti-apoptotic factors except for Bcl-2 differ significantly during the change in LMAN size, indicating that Bcl-2 is probably responsible for these changes.

Consistent with the fact that no obvious decreases in the sizes of the song nuclei were observed (with the exceptions of the female RA and the male and female LMAN), no significant gender-related differences in the densities of caspase-3 or other pro- or anti-apoptotic factors were observed in the other nuclei. In addition, we noted that two pro-apoptotic factors (Bid and Bax) and one anti-apoptotic factor (Bcl-2) were expressed at much higher levels in the male HVC than in the female HVC. We also noted that two pro-apoptotic members (Bid and Bax) and one anti-apoptotic member (Bcl-2) were expressed at much higher levels in the male HVC than in the female HVC. Considering that cell apoptosis might also be determined by the ratio of anti-apoptotic to pro-apoptotic protein [21, 58, 59], sexual differences in the expression of Bid, Bax, or Bcl-2 in the HVC might not definitively lead to apoptosis. It should also be considered that active caspase-3 and pro- or anti-apoptotic factors might be involved in activities other than apoptosis, such as cell cycle regulation, cell proliferation and differentiation [60], and neuron survival [60–63]. These reports are helpful to explain why caspase-3 and some pro- and anti-apoptotic members were detected in adult song nuclei that did not exhibit changes in size. Additionally, these reports are useful for understanding the potential roles of the dynamic distributions observed for the above cell apoptosis-related factors in song control nuclei.

### Neural mechanism of the decreases in size of song control nuclei

Although the RA is similar in males and females prior to the critical period (around P30), its neuron number, volume and size decrease to a much greater extent in females than in males [13, 14]. Unlike the HVC or Area X, almost none of the newborn neurons generated in the ventricular zone migrate to the RA after hatching [9]. Thus, cell loss in the female RA is the sole reason for the difference between the sexes. Although many studies have addressed this issue, the mechanism of this neuronal reduction remains unclear.

The RA receives afferent input from the LMAN and HVC. Unilateral HVC lesions at P20 increased cell death and decreased neuron number and soma size within the ipsilateral RA in both sexes. In contrast, unilateral LMAN lesions or simultaneous LMAN and HVC lesions at the same age caused more pronounced decreases in the number of RA neurons [5, 11]. An additional study has revealed that direct infusions of neurotrophins, including BDNF, into the RA completely suppress RA apoptosis after LMAN injury [40]. In the present study, we first compared the densities of cells expressing BDNF and TrkB in the song control nuclei. Although the densities of BDNF- and TrkB-positive cells did not exhibit sexual differences in the majority of the studied groups, the total number of BDNF- and TrkB-positive neurons in the song nuclei were greater in males than in females (data not shown; the volumes of the song control nuclei were larger in males than in females, with the exception of the LMAN after P35). These reports are consistent with previous reports showing the dynamic expression of BDNF and TrkB in song control nuclei around the critical period (P30) [64, 65]. We further found that RA volume and the total number of TrkB-positive cells in the RA decreased more markedly following unilateral LMAN lesion than unilateral HVC lesion (Fig 11G). Additionally, we found that the total number of BDNF-positive cells that project to the RA from the LMAN was greater than the number from the HVC (Fig 11H). Regarding reports that BDNF is involved in cell survival [40, 41], our results showed that the difference in the total number of RA-projecting cells that were immunoreactive for BDNF between LMAN and HVC might explain previous data showing more serious damage in the RA after LMAN lesion than after HVC lesion [5, 11]. Fewer RA-projecting cells have been reported in the female HVC [66], which might lead to the transport of less BDNF to the female RA, resulting in greater cell loss in the females than in males.

Thus, the present study provides data addressing how Bcl-2 family members (both pro- and anti-apoptotic) are involved in apoptosis in song control nuclei and how BDNF might contribute to the sexual dimorphism of song control nuclei of songbirds.
